# Exhaustion of CD8^+^ central memory responder T cell differentiation provokes non-melanoma skin cancer in elderly kidney transplant recipients

**DOI:** 10.3389/fimmu.2023.1164284

**Published:** 2023-05-23

**Authors:** Jonas Leonhard, Matthias Schaier, Florian Kälble, Martin Zeier, Andrea Steinborn

**Affiliations:** ^1^ Department of Obstetrics and Gynecology, University of Heidelberg, Heidelberg, Germany; ^2^ Department of Nephrology, University of Heidelberg, Heidelberg, Germany

**Keywords:** kidney transplantation, non-melanoma skin cancer, immunosuppressive therapy, CD8+ T cell differentiation, CD8+ regulatory T cells, CD8+ responder T cells, CD8+ T cell exhaustion, cancer immunity

## Abstract

**Introduction:**

Immunosuppressive therapy prevents graft rejection but increases the risk of non-melanoma skin cancer (NMSC), especially in elderly kidney transplant recipients (KTR).

**Methods:**

In this study, we separately investigated the differentiation of CD8^+^ regulatory T cells (Tregs) and responder T cells (Tresps) between healthy KTR without NMSC, KTR developing *de-novo* NMSC within two years after the enrolment, and KTR with NMSC at the time of enrolment. Antigen-unexperienced CCR7^+^CD45RA^+^CD31^+^ recent thymic emigrant (RTE) cells differentiate *via* CD45RA^-^CD31^+^ memory (CD31^+^ memory) cells, *via* resting mature naïve (MN) cells or *via* direct proliferation into CD45RA^-^CD31^-^ memory (CD31^-^ memory) cells, consisting of both CCR7^+^CD45RA^-^ central memory (CM) and CCR7^-^CD45RA^-^ effector memory (EM) cells.

**Results:**

We found that both RTE Treg and Tresp differentiation *via* CD31^+^ memory Tregs/Tresps was age-independently increased in KTR, who developed *de novo* NMSC during the follow-up period, causing abundant CM Treg/Tresp production, which may be crucial for cancer immunity. These changes favored a strongly increased CD8^+^ Treg/Tresp ratio, suggesting this ratio as a reliable marker for *de-novo* NMSC development in KTR. However, with age, this differentiation was replaced by increased conversion of resting MN Tregs/Tresps into CM Tregs/Tresps, which exhausted for Tresps but not for Tregs. In KTR with already existing NMSC at enrolment, differentiation was maintained *via* conversion and proliferation of resting MN Tregs/Tresps, which however increasingly exhausted with age, especially for Tresps. This resulted in a strong accumulation of terminally differentiated effector memory (TEMRA) Tresps in elderly individuals. Patients with NMSC recurrence showed increased proliferation of resting MN Tregs/Tresps into EM Tregs/Tresps, which tended to exhaust more rapidly, particularly for Tresps, than in patients without NMSC recurrence.

**Discussion:**

In conclusion, we provide evidence that immunosuppressive therapy inhibits differentiation of CD8^+^ Tregs more than that of CD8^+^ Tresps, resulting in an exhausted Tresp profile, thus providing a possible therapeutic approach to improve poor cancer immunity in elderly KTR.

## 1 Introduction

Kidney transplantation is the preferred therapy of patients with kidney failure since it reduces mortality and improves the quality of life compared to dialysis treatment (1, 2). However, transplantation requires immunosuppressive therapy, which increases the risk of malignancy approximately three times in kidney transplant recipients (KTR) (3). Non-melanoma skin cancer (NMSC) represents the most common malignancy with an up to 250-fold increased incidence among long-term KTR compared to the healthy population (4). Thereby, post-transplant skin cancer appears to be more aggressive with higher rates of metastasis, synchronous internal malignancies, and overall mortality (5–7).

Adaptive immunity plays a crucial role in the response to NMSC (8). Especially CD8^+^ T cells, which similarly like CD4^+^ T helper cells consist of responder T cells (Tresps) and regulatory T cells (Tregs), are essential for the effective control of tumor growth (9, 10). Both the Treg and Tresp pool contain CCR7^+^CD45RA^+^ naïve cells, which were shown to differentiate into CCR7^+^CD45RA^-^ central memory (CM), CCR7^-^CD45RA^-^ effector memory (EM), and CCR7^-^CD45RA^+^ terminally differentiated effector memory (TEMRA) cells. CM cells with various immune-stimulatory functions are considered as progenitor effector cells, whereas EM and TEMRA cells are thought to represent fully differentiated effector subsets (11). Moreover, the naïve CCR7^+^CD45RA^+^ Treg/Tresp pool includes CD45RA^+^CD31^+^ recent thymic emigrants (RTEs), which can differentiate *via* CD45RA^-^CD31^+^ memory (CD31^+^ memory) cells or *via* CD45RA^+^CD31^-^ mature naïve (MN) cells into CD45RA^-^CD31^-^ memory (CD31^-^ memory) Treg/Tresp cells. Thereby, MN cells seem to function as long-living reserve population in the naïve cell pool, preserving differentiation in case of RTE exhaustion (12).

Chronic kidney failure is known to affect the T cell compartment, favoring accelerated aging of the distributed T cells (13–15) and thereby even reducing survival after kidney transplantation (16, 17). Recently, we showed that chronic kidney failure provokes an increased CD8^+^ Tresp differentiation with the accumulation of TEMRA Tresps and reduction of CM Tresp differentiation after transplantation (18). In this regard, CM Tresps were shown to be crucial for effective immune stimulation and early immune response, while accumulating TEMRA Tresps exhibited limited cytotoxicity, which may further increase the cancer susceptibility of immunosuppressed KTR.

Clinical risk factors for post-transplant skin cancer, such as pre-transplant skin cancer, increased age, and male sex, are well established (19). Additionally, the immunosuppressive regimen influences cancer incidence as mammalian target of rapamycin inhibitors prevent tumor growth by anti-angiogenesis (20). On the other hand, calcineurin inhibitors and purine analogues have pro-oncogenic properties through various mechanisms (8). Several approaches were made to identify immunological risk factors for post-transplant skin cancer. First, high numbers of regulatory CD4^+^FoxP3^+^CD127^low+^ Tregs (21) or CD4^+^C25^+^ T cells (22) were found to correlate with the occurrence of NMSC in KTR. Later, it became apparent that rather a low number of CD8^+^ T cells and a high CD4^+^/CD8^+^ T cell ratio may indicate the development of NMSC (23). Therefore, the differential influence of immunosuppressive therapy on the differentiation of CD8^+^ Tregs and Tresps, respectively, could be of critical importance. Currently, little is known about the role of CD8^+^ Treg and CD8^+^ Tresp differentiation in tumor pathogenesis after transplantation.

Our study reveals differences in the differentiation of CD8^+^ RTE Tregs/Tresps into CD31^-^ memory Tregs/Tresps between healthy KTR without NMSC, KTR who developed *de-novo* NMSC during a follow-up period of two years after the measurement, and KTR with already existing NMSC at enrolment. We showed that both RTE Treg and Tresp differentiation *via* CD31^+^ memory Tregs/Tresps was age-independently increased in KTR who developed NMSC during the follow-up period, causing strong CM Treg/Tresp production. These changes were associated with a greatly increased CD8^+^ Treg/Tresp ratio, proposing this ratio as a reliable marker for *de-novo* NMSC development in KTR. However, intensified RTE Treg/Tresp differentiation could not be maintained in KTR with apparent NMSC at enrolment. Our data support the hypothesis that only Tregs but not Tresps were able to replenish the CD31^-^ memory Treg/Tresp pool with age by RTE Treg differentiation *via* resting MN Tregs. In conclusion, our findings suggest limited cancer immunity, especially in elderly KTR with a history of cancer.

## 2 Material and methods

### 2.1 Study participants

Blood samples were collected from 133 KTR without NMSC and 55 KTR with a history of NMSC during routine visits at the department of nephrology, University of Heidelberg. Participants had undergone kidney transplantation at least three months before enrolment and did not suffer from a recent illness or autoimmune disease. Exclusion criteria for KTR without NMSC were an elevated C-reactive protein > 5 mg/l, serum creatinine > 2 mg/dl, or a history of malignancy. Participants with NMSC at enrolment developed NMSC or a precursor lesion (squamous cell carcinoma, actinic keratosis, Bowen disease, or basal cell carcinoma) after transplantation under immunosuppressive therapy. KTR with a history of pre-transplant skin cancer were excluded.

We then followed the incidence of skin cancer up in the healthy group without NMSC after a median of 2.1 (0.5 - 2.7) years from enrolment. Two patients died shortly after the measurement, one patient returned to dialysis due to chronic graft failure, and one patient was not reachable for follow-up. Six patients developed NMSC shortly after the enrolment: two patients with squamous cell carcinoma, actinic keratosis, and Bowen disease, one patient each with basal cell carcinoma, Bowen disease, and actinic keratosis, and one patient with actinic keratosis and basal cell carcinoma. **Table 1** reveals the clinical data of all participants at enrolment.

**Table 1:** Clinical characteristics of the study participants.

	Healthy KTR	KTR with NMSC	KTR developing NMSC
	n = 123	n = 55	n = 6
Female	53 (43)	15 (28)	2 (33)
Age (y)	52 (23-82)	66 (35-82)	53.5 (41-71)
Time on dialysis (y)	2.7 (0-12.8)	3.6 (0-14.7)	1.9 (0-9.1)
Deceased donor kidney	63 (51)	36 (67)	2 (33)
Time since transplantation (y)	6.1 (0.2-28.4)	12.8 (3.8-35.9)	4.0 (0.4-8.0)
Treated for graft rejection	41 (33)	28 (52)	1 (17)
Immunosuppression			
Tac + MPA + Steroid	40 (33)	17 (31)	3 (50)
CsA + MPA + Steroid	52 (42)	14 (26)	2 (33)
mTOR-inh. + MPA + Steroid	6 (5)	4 (7)	1 (17)
Azathioprine + others	4 (3)	5 (9)	0 (0)
Belatacept + others	2 (2)	4 (7)	0 (0)
Others	19 (15)	10 (19)	0 (0)
NMSC			
Squamous cell carcinoma		24 (44)	2 (33)
Actinic keratosis		18 (33)	4 (67)
Bowen disease		16 (30)	3 (50)
Basal cell carcinoma		32 (59)	2 (33)
Serum creatinine (mg/dl)	1.26 (0.63-1.90)	1.36 (0.60-2.83)	1.15 (0.88-1.75)
Urea (mg/dl)	40 (14-84)	58 (15-153)	37 (25-66)
CKD-EPI GFR (ml/min/1.73m²)	60.9 (32.3-114.8)	52.8 (22.7-97.7)	66.3 (39.7-84.8)
C-reactive protein (mg/l)	<2.0 (<2.0-4.7)	2.2 (<2.0-32.8)	<2.0 (<2.0-<2.0)

Categorial variables are presented as number (percentage), continuous variables as median (minimum-maximum). CKD-EPI GFR, chronic kidney disease epidemiology collaboration estimated glomerular filtration rate; CsA, ciclosporin A; KTR, kidney transplant recipients; MPA, mycophenolic acid; mTOR-inh., mechanistic target of rapamycin-inhibitor; NMSC, non-melanoma skin cancer; Tac, tacrolimus.

During a follow-up appointment 2.0 (1.0 – 3.1) years after the measurement, we asked about the recurrence of NMSC. Of 55 patients at enrolment, two patients died before follow-up, two patients had chronic graft failure and returned to dialysis, and two participants were unavailable for follow-up. Of the remaining 49 patients developed 24 patients recurrent skin cancer, while 25 KTR remained skin cancer free.

The Regional Ethics Committee approved the study (reference number S-523/2012, 05.07.2018). All participants were fully informed about the study settings and aims. Written informed consent was received from all individuals.

### 2.2 Fluorescence-activated cell sorting staining

Nine ml of venous blood were collected into ethylenediaminetetraacetic acid (EDTA)-containing tubes. Subsequently, peripheral blood mononuclear cells (PBMCs) were isolated *via* density gradient centrifugation using Lymphodex (Inno-Train Diagnostik GmbH, Kronberg, Germany) according to the manufacturer’s instructions. PBMCs (8 x 10^6^) were then surface-stained with 10 µl peridinin-chlorophyll-protein (Per CP)-conjugated anti-CD8 (BD Biosciences, Heidelberg, Germany), 5µl phycoerythrin-cyanine 7 (PE-Cy7)-conjugated anti-CD127 (eBioscience, Frankfurt, Germany), 5 µl phycoerythrin (PE)-conjugated anti-CCR7 (Biolegend, San Diego, USA), 5 µl allophycocyanin-H7 (APC-H7)-conjugated anti-CD45RA (BD Biosciences), and 5 µl Alexa Fluor 647-conjugated anti-CD31 (BD Biosciences) undiluted mouse monoclonal antibodies according to the manufacturer’s instructions. After 20 minutes of incubation at 6 °C, PBMCs were washed twice with 3 ml phosphate-buffered saline (PBS) and centrifuged with 483 g for 5 minutes. Intracellular forkhead box P3 (FoxP3) was detected using a fluorescein isothiocyanate (FITC)-conjugated anti-human FoxP3 staining set (clone PCH101, eBioscience) according to the manufacturer’s instructions and washed twice after the staining. Negative control samples were incubated with isotype-matched antibodies. Cells were analyzed with a FACS Canto cytometer (BD Biosciences) immediately after the staining. Dead cells and doublets were excluded *via* forward and side scatter characteristics (FSC and SSC). Statistical analysis was based on at least 100.000 CD8^+^ T cells.

### 2.3 Gating strategy, cell subsets, and differentiation pathways

**Figure 1:**
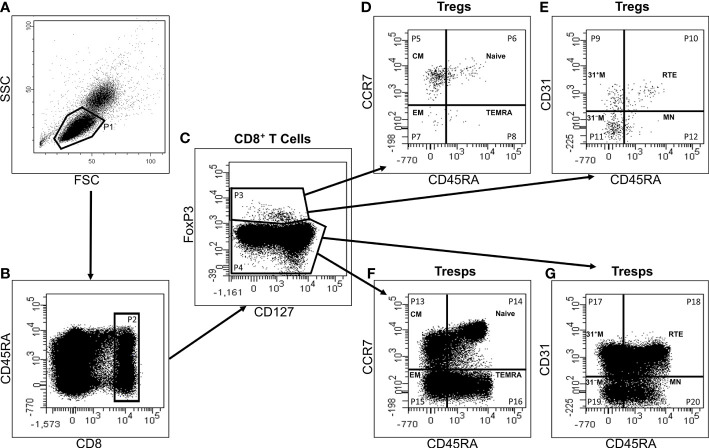
Gating strategy for six-color-flow-cytometric division of CD8^+^CD127^low+/-^FoxP3^+^ Tregs and CD8^+^CD127^+/-^FoxP3^-^ Tresps into their subsets. First, all lymphocytes (P1) were detected by side scatter characteristics (SSC) versus forward scatter characteristics (FSC) **(A)**. Then, we examined the fluorescence activity of CD8 versus CD45RA **(B)** to determine the percentage of CD8^+^ T cells of all lymphocytes (P2). Afterwards, the fluorescence activity of FoxP3 versus CD127 was presented **(C)** to separate CD8^+^ Tregs (P3) from Tresps (P4). Finally, Tregs and Tresps were separately divided into naïve (P6, P14), CM (P5, P13), EM (P7, P15), and TEMRA (P8, P16) cells, respectively, by analyzing the fluorescence activity of CCR7 versus CD45RA **(D, F)**. The percentage of RTE (P10, P18), MN (P12, P20), CD31^+^ memory (P9, P17) and CD31^-^ memory (P11, P19) Tregs/Tresps was identified by using fluorescence activity of CD31 versus CD45RA **(E, G)**.

**Figure 1** exhibits the gating strategy for these measurements. We determined the percentage of CD8^+^ T cells of all lymphocytes and separated them into CD8^+^CD127^low+/-^FoxP3^+^ Tregs and CD8^+^CD127^+/-^FoxP3^-^ Tresps (**Figures 1A–C**). Then, we divided both CD8^+^ Tregs and Tresps into CD45RA^+^CCR7^+^ naïve, CD45RA^-^CCR7^+^ CM, CD45RA^-^CCR7^-^ EM, and CD45RA^+^CCR7^-^ TEMRA Tregs/Tresps (**Figures 1D, F**) and into CD45RA^+^CD31^+^ RTE, CD45RA^+^CD31^-^ resting MN, CD45RA^-^CD31^+^ memory and CD45RA^-^CD31^-^ memory Tregs/Tresps (**Figures 1E, G**). Finally, we merged these two differentiation schemata to distinguish newly released antigen-unexperienced CCR7^+^CD45RA^+^CD31^+^ RTE cells (RTEs) from CCR7^-^CD45RA^+^CD31^+^ TEMRA cells (CD31^+^ TEMRAs) and CCR7^+^CD45RA^+^CD31^-^ resting MN cells (resting MNs) from CCR7^-^CD45RA^+^CD31^-^ TEMRA cells (CD31^-^ TEMRAs).

This enabled us to recognize three different differentiation pathways of CD8^+^ RTE T cells. Firstly, they can differentiate *via* CD31^+^ memory cells into CD31^-^ memory cells (pathway 1) and might produce CD31^+^ TEMRA cells when this pathway is exhausted. Secondly, RTE T cells can directly proliferate into CD31^-^ memory cells (pathway 2), whereby CD31^-^ TEMRA cells might emerge when this proliferation exhausts. Thirdly, RTE T cells can proliferate into resting MN cells, which subsequently might convert into CD31^-^ memory cells or proliferate into CD31^-^ memory cells (pathway 3). Thereby, it seems that CM cells predominantly arise *via* pathway 1, while EM cells rather emerge *via* pathway 2. Resting MN cells may be able to convert or proliferate into CD31^-^ memory cells, producing both CM and EM cells.

### 2.4 Statistical analysis

We used linear regression to examine changes in the percentage of CD8^+^ T cells, Tregs, Tresps, and their subsets during the course of life using separate models for each patient group. R^2^ coefficients of determination of the linear regressions are provided in **Supplementary Table 1**. Age-independent group differences were examined using multiple regression analysis adjusted for the age variable (centered on the mean), wherein an interaction term of the age and the patient group was included. A p-value < 0.05 was considered significant. However, this research is an exploratory study in which the calculated p-values are descriptive, but not confirmatory. For all statistical tests, the software package BiAS for Windows (version 10.06) was used. For data presentation, we used Microsoft Office (version 2212) and GraphPad Prism 9.

## 3 Results

### 3.1 With age, kidney transplant recipients suffering from non-melanoma skin cancer maintain their memory Treg pool by increased differentiation of resting MN Tregs

**Figure 2:**
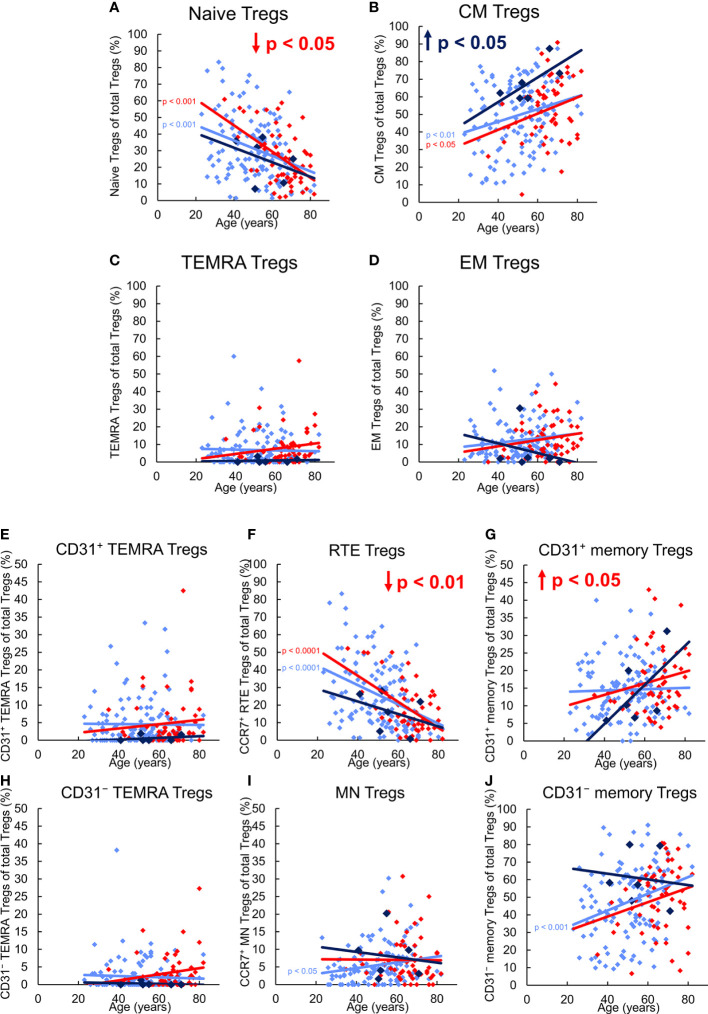
Differentiation of CD8^+^ Tregs in KTR without NMSC (n = 123), KTR with NMSC at enrolment (n = 55), and KTR developing NMSC for the first time during the follow-up period (n = 6). The figures present the percentage of naïve **(A)**, CM **(B)**, TEMRA **(C)**, and EM **(D)** Tregs within total Tregs of KTR without NMSC in light blue (♦), KTR with NMSC at enrolment in red (♦), and KTR developing NMSC during the follow-up period in dark blue (♦). To recognize different differentiation pathways, the figure additionally shows the proportion of CD31^+^ TEMRA **(E)**, RTE **(F)**, CD31^+^ memory **(G)**, CD31^-^ TEMRA **(H)**, MN **(I)**, and CD31^-^ memory Tregs **(J)** within total Tregs. Color-matched regression lines reveal changes with age, respectively, whereby significant changes are indicated by the p-value in front of the regression line. Age-independent significant differences of KTR with NMSC at enrolment or KTR developing NMSC during the follow-up period compared to those without NMSC are marked by an arrow and their color-matched p-values.

**Figure 3:**
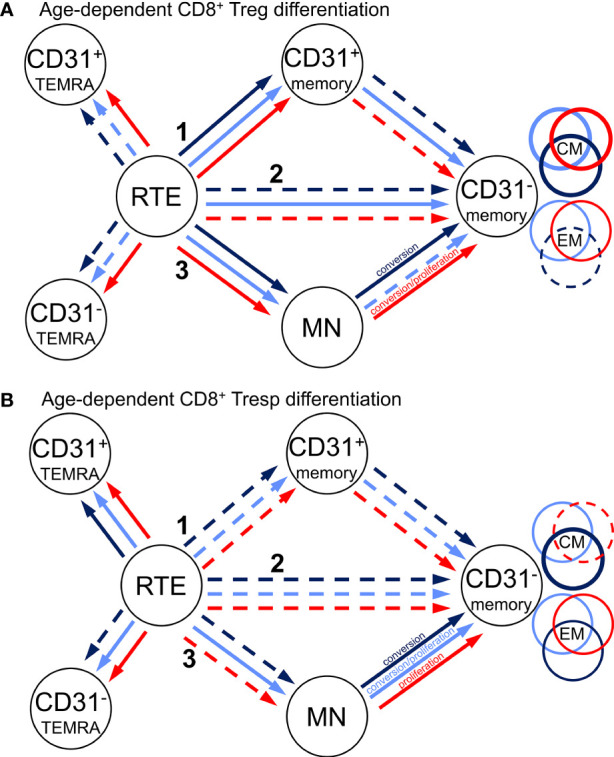
Proposed age-dependent differentiation pathways of CD8^+^ Tregs **(A)** and Tresps **(B)** for KTR without NMSC (light blue), KTR with NMSC at enrolment in (red), and KTR developing NMSC for the first time during the follow-up period (dark blue). RTE cells can differentiate *via* CD31^+^ memory cells (1), directly proliferate (2), or differentiate *via* MN cells (3) into CD31^-^ memory cells. Color-matched circles show if CD31^-^ memory cells consist of CM or EM cells.

**Figure 2** reveals differences in the differentiation of CD8^+^ Treg cells between 123 healthy KTR without NMSC, six KTR developing NMSC for the first time during the follow-up period, and 55 patients who already had NMSC at enrolment. In healthy KTR, with age, the percentage of naïve Tregs decreased significantly within the total Treg pool, while that of CM and EM Tregs increased, whereby significance was reached for CM, but not EM Tregs (**Figures 2A, B, D**). In contrast, the percentage of TEMRA Tregs remained unchanged (**Figure 2C**). Further division of the naïve Treg pool into RTE Tregs, MN Tregs, CD31^+^, and CD31^-^ TEMRA Tregs revealed significantly decreasing RTE Tregs (**Figure 2F**), but increasing resting MN and CD31^-^ memory Tregs (**Figures 2I, J**), while CD31^+^ and CD31^-^ TEMRA Tregs, and CD31^+^ memory Tregs did not change (**Figures 2E, G, H**). These results suggest that during the course of life, RTE Tregs differentiate *via* CD31^+^ memory Tregs (pathway 1) or proliferate (pathway 2) into CD31^-^ memory Tregs. Obviously, this differentiation produces mainly CM, but less EM Tregs, whereas resting MN Tregs are enriched as a naïve reserve population (**Figure 3A**).

Compared to healthy KTR without NMSC, those who developed NMSC during the follow-up period for the first time exhibited a significantly increased proportion of CM Tregs within the total Treg pool independent of age (**Figure 2B**), presumably due to increased RTE Treg differentiation *via* CD31^+^ memory Tregs into CD31^-^ memory Tregs (pathway 1). This may be attributed to a lower proportion of RTE Tregs, a higher proportion of resting MN Tregs, and a higher proportion of CD31^-^ memory Tregs within the total Treg pool (**Figures 2F, I, J**). However, no significance was reached because of the small number of patients in this group. As there was no age-independent CD31^+^ or CD31^-^ TEMRA Treg accumulation in this patient group (**Figures 2E, H**), RTE Treg differentiation *via* CD31^+^ memory Tregs (pathway 1) or their proliferation (pathway 2) does not seem to be affected. However, with age, the percentage of CM Tregs further increased, while that of EM Tregs rather decreased (**Figures 2B, D**) although RTE Treg differentiation *via* CD31^+^ memory Tregs does not seem to be maintainable, as the proportion of CD31^+^ memory Tregs increased strongly within the total Treg pool (**Figure 2G**). In contrast, the percentage of resting MN Tregs no longer increased in these patients (**Figure 2I**), suggesting that these cells were used to replenish the CD31^-^ memory Treg pool with age (pathway 3). Presumably, conversion but not proliferation of these cells ensured an increasing CM but decreasing EM Treg production with age (**Figure 3A**).

In KTR already suffering from NMSC at the time of enrolment, the percentage of naïve Tregs within the total Treg pool was age-independently diminished compared to those participants without NMSC although that of CM, EM, and TEMRA Tregs was not different (**Figures 2A-D**). Further subdivision of the naïve Treg pool revealed that the percentage of RTE Tregs was significantly decreased while that of CD31^+^ or CD31^-^ TEMRA Tregs (**Figures 2E, H**) as well as that of MN Tregs (**Figure 2I**) remained unchanged. As the percentage of CD31^+^ memory Tregs was significantly increased in KTR with NMSC (**Figure 2G**), while the percentage of CD31^-^ memory Tregs was preserved, it seems that the impairment of RTE Treg differentiation *via* CD31^+^ memory Tregs was compensated by an increased differentiation *via* proliferation of RTE Tregs and conversion of resting MN Tregs (pathway 2 and 3). Thereby the proportions of CM and EM Tregs within the total Treg pool could be maintained. With age, these patients accordingly revealed exhausted RTE Treg differentiation *via* CD31^+^ memory Tregs, as there was an increase of CD31^+^ TEMRA Tregs and CD31^+^ memory Tregs (**Figures 2E, G**). Since the percentage of CD31^-^ TEMRA Tregs also increased with increasing age (**Figure 2H**), RTE Treg proliferation might additionally be affected. This means that with age, KTR with NMSC replenish their CD31^-^ memory Treg pool of CM and EM Tregs through proliferation and conversion of resting MN Tregs (pathway 3) (**Figure 3A**). Such findings suggest that KTR cannot maintain an age-independently increased CM Treg production until NMSC is diagnosed, but eventually reveal intensified RTE Treg differentiation *via* resting MN Tregs to maintain an increasing CD31^-^ memory Treg pool with age.

### 3.2 With age, kidney transplant recipients suffering from non-melanoma skin cancer cannot maintain their memory Tresp pool, due to exhaustion of resting MN Tresp differentiation

**Figure 4:**
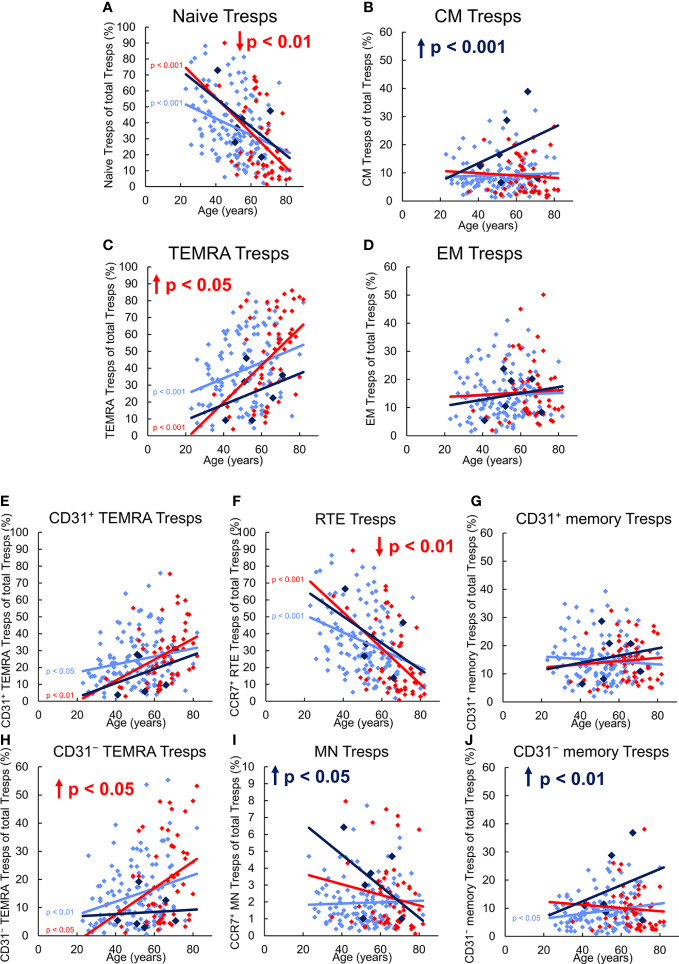
Differentiation of CD8^+^ Tresps in KTR without NMSC (n = 123), KTR with NMSC at enrolment (n = 55), and KTR developing NMSC for the first time during the follow-up period (n = 6). The figures present the percentage of naïve **(A)**, CM **(B)**, TEMRA **(C)**, and EM **(D)** Tresps within total Tresps of KTR without NMSC in light blue (♦), KTR with NMSC at enrolment in red (♦), and KTR developing NMSC during the follow-up period in dark blue (♦). To recognize different differentiation pathways, the figure additionally shows the proportion of CD31^+^ TEMRA **(E)**, RTE **(F)**, CD31^+^ memory **(G)**, CD31^-^ TEMRA **(H)**, MN **(I)**, and CD31^-^ memory Tresps **(J)** within total Tresps. Color-matched regression lines reveal changes with age, respectively, whereby significant changes are indicated by the p-value in front of the regression line. Age-independent significant differences of KTR with NMSC at enrolment or KTR developing NMSC during the follow-up period compared to those without NMSC are marked by an arrow and their color-matched p-values.

**Figure 4** shows differences in the differentiation of CD8^+^ Tresp cells between these three patient groups. In healthy KTR, with age, the percentage of naïve Tresps decreased, while that of TEMRA Tresps increased significantly. In contrast, the percentages of CM and EM Tresps increased only slightly (**Figures 4A-D**). As the percentages of both CD31^+^ and CD31^-^ TEMRA Tresps also increased significantly (**Figures 4E, H**), RTE Tresp differentiation *via* CD31^+^ memory Tresps and direct proliferation into CD31^-^ memory Tresps seems to be increasingly exhausted with age (pathway 1 and 2). Instead, RTE Tresps might differentiate *via* MN Tresp conversion and proliferation (pathway 3) to maintain an increasing CD31^-^ memory Tresp pool of both CM and EM Tresps with age (**Figure 3B**).

Independent of age, KTR developing NMSC during the follow-up period revealed a significantly increased percentage of CM Tresps, MN Tresps, and CD31^-^ memory Tresps within the total Tresp pool (**Figures 4B, I, J**). These differences suggest an intensified differentiation of RTE Tresps *via* CD31^+^ memory Tresps into CD31^-^ memory Tresps and enrichment of resting MN Tresps as naïve reserve population (pathway 1), similarly as observed for Treg differentiation. A lack of CD31^+^ or CD31^-^ TEMRA Tresp enrichment (**Figures 4E, H**) also proposes that there is no impairment of RTE Tresp differentiation or proliferation. With age, the percentage of naïve Tresps was decreasing and that of CM, TEMRA, and EM Tresps increasing with a noticeable increase of CM Tresps (**Figures 4A–D**). Thereby, the percentage of CD31^+^ TEMRA Tresps and CD31^+^ memory Tresps also increased with age (**Figures 4E, G**), indicating exhausted RTE Tresp differentiation *via* CD31^+^ memory Tresps after age-independently intensified differentiation *via* that pathway. The proportions of RTE Tresps and MN Tresps declined, while that of CD31^-^ memory Tresps increased with age (**Figures 4F, I, J**), suggesting RTE Tresp differentiation *via* resting MN Tresp conversion (pathway 3) into CD31^-^ memory Tresps instead (**Figure 3B**).

In KTR with NMSC at enrolment, the percentage of naïve Tresps was significantly decreased, while that of TEMRA Tresps was increased compared to KTR without NMSC (**Figures 4A, C**), implying age-independently intensified RTE Tresp proliferation into TEMRA Tresps instead of CM or EM Tresps. Further subdivision shows that the percentage of RTE Tresps was significantly decreased and that of CD31^-^ TEMRA Tresps increased (**Figures 4F, H**), indicating exhausted RTE Tresp proliferation into CD31^-^ memory Tresps. With age, KTR with NMSC at enrolment had a decreasing percentage of naïve and CM Tresps and an increasing percentage of TEMRA and EM Tresps (**Figures 4A-D**), although significance was only achieved for naïve and TEMRA Tresps. Thus, these patients cannot maintain an increasing CM Tresp pool with age and strongly accumulate TEMRA Tresps instead. As the percentage of CD31^+^ and CD31^-^ TEMRA Tresps significantly increased and that of CD31^+^ memory Tresps slightly increased with age (**Figures 4E, G, H**), both RTE Tresp differentiation *via* CD31^+^ memory Tresps and direct RTE proliferation into CD31^-^ memory Tresps seems to be diminished with age (**Figure 3B**). Thereby, the percentage of RTE Tresps, MN Tresps, and CD31^-^ memory Tresps decreased with age (**Figures 4F, I, J**), which means that the remaining MN Tresps might proliferate into CD31^-^ memory Tresps to produce EM Tresps but cannot convert into CD31^-^ memory Tresps to replenish CM Tresps with age (pathway 3). The decreasing percentage of CD31^-^ memory Tresps reflects that Tresp differentiation seems to strongly be limited especially in elderly patients with a history of NMSC.

### 3.3 Increased CD8^+^ Treg/Tresp ratio in kidney transplant recipients could predict *de-novo* non-melanoma skin cancer

**Figure 5:**
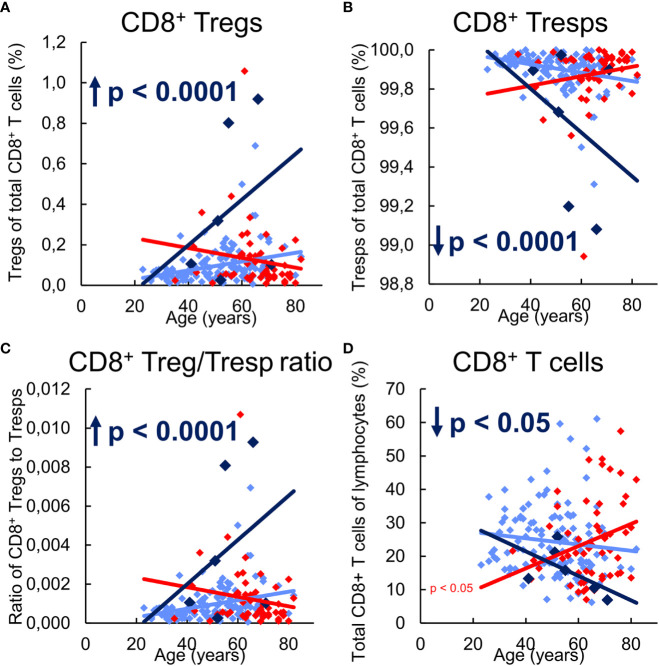
Composition of CD8^+^ T cells with CD8^+^CD127^low+/-^FoxP3^+^ Tregs and CD8^+^CD127^+/-^FoxP3^-^ Tresps in KTR without NMSC (n = 123), KTR with NMSC at enrolment (n = 55), and KTR developing NMSC for the first time during the follow-up period (n = 6) and its influence on their Treg/Tresp ratio and the percentage of total CD8^+^ T cells. The diagrams exhibit the proportion of Tregs **(A)** and Tresps **(B)** within total CD8^+^ T cells, the CD8^+^ Treg/Tresp ratio **(C)**, and the percentage of total CD8^+^ T cells of all lymphocytes **(D)** of KTR without NMSC in light blue (♦), KTR with NMSC at enrolment in red ♦), and KTR developing NMSC during the follow-up period in dark blue (♦). Color-matched regression lines reveal changes with age, respectively, whereby significant changes are indicated by the p-value in front of the regression line. Age-independent significant differences of KTR with NMSC at enrolment or KTR developing NMSC during the follow-up period compared to those without NMSC are marked by an arrow and their color-matched p-values.

In the following, we investigated the influence of the differing differentiation in the three study groups on the composition of the total CD8^+^ T cell pool with CD8^+^ Tregs or CD8^+^ Tresps, their ratio to each other, and on the proportion of total CD8^+^ T cells in all lymphocytes. In healthy KTR without NMSC, there was a slight increase of CD8^+^ Tregs with a decrease of Tresps and thus an increasing CD8^+^ Treg/Tresp ratio with rather decreasing CD8^+^ T cells, presumably due to increased differentiation into CD31^-^ memory Tregs, but TEMRA Tresps with age (**Figures 5A-D**).

KTR developing NMSC during the follow-up period revealed a significantly increased percentage of CD8^+^ Tregs and a complementarily decreased percentage of CD8^+^ Tresps, leading to an increased Treg/Tresp ratio and a significantly decreased percentage of CD8^+^ T cells compared to KTR without NMSC (**Figures 5A-D**). These findings appear to be based on an age-dependent development of these age-independent changes. Thereby, the increasing conversion of resting MN Tregs/Tresp into CM Tregs/Tresps seems to exhaust for Tresps but not for Tregs through which the percentage of CD8^+^ T cells within total lymphocytes, as well as the percentage of CD8^+^ Tresps within total CD8^+^ T cells decrease with age. This causes an increasing Treg/Tresp ratio with age in KTR developing NMSC during the follow-up period. The also age-independently increased CD8^+^ Treg/Tresp ratio could represent a novel approach to detect *de-novo* NMSC in KTR before its clinical occurrence.

Independent of age, KTR with NMSC did not reveal any differences in the percentage of CD8^+^ Tregs, Tresps, their Treg/Tresp ratio, or the percentage of total CD8^+^ T cells compared to KTR without NMSC (**Figures 5A-D**). With age, these patients exhibited a declining proportion of CD8^+^ Tregs, while that of Tresps inclined so that the CD8^+^ Treg/Tresp ratio declined, although significance was not reached. However, their percentage of total CD8^+^ T cells significantly increased with age, suggesting fundamental differences in the age-dependent CD8^+^ T cell differentiation between KTR with and without NMSC. This contrasting age-dependent development might be caused by the exhausted MN Tresp differentiation into CD31^-^ memory Tresps in KTR with NMSC which favored proliferation instead of conversion of MN Tresps into CD31^-^ memory Tresps.

### 3.4 NMSC recurrence is associated with exhausting proliferation of resting MN Tresps into EM Tresps

**Figure 6:**
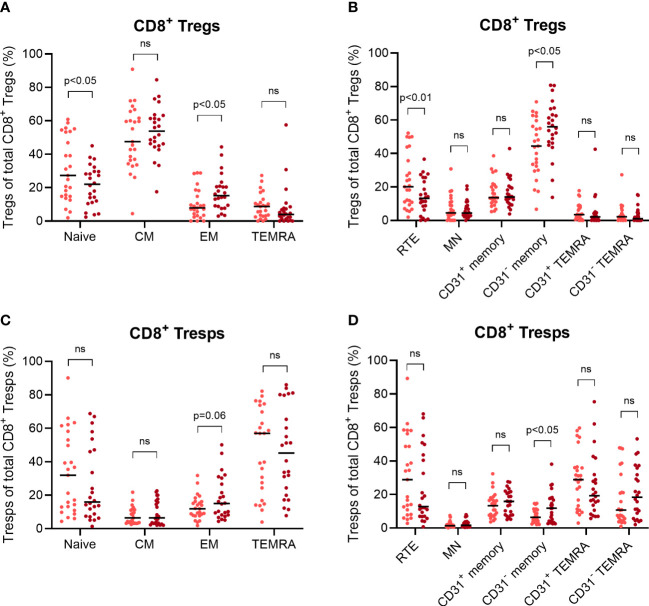
Differences in the age-independent differentiation of CD8^+^ Tregs and Tresps between KTR with NMSC at enrolment who did not develop recurrent cancer during the follow-up period (n = 25) and KTR with recurrent cancer during the follow-up period (n = 24). The diagrams reveal the percentage of naïve, CM, EM, and TEMRA Tregs/Tresps **(A, B)** as well as the percentage of RTE, MN, CD31^+^ memory, CD31^-^ memory, CD31^+^ TEMRA, and CD31^-^ TEMRA Tregs/Tresps **(C, D)** of total Tregs/Tresps, with the median as a bar for KTR without recurrent NMSC (light red) and for KTR with recurrent NMSC (dark red). Significant age-independent differences are indicated by the respective p-value. ns, not significant.

Finally, we examined NMSC recurrence in those KTR who already had NMSC at enrolment to recognize differences in their CD8^+^ Treg and Tresp differentiation. After a follow-up period of two years, 25 KTR did not develop recurrent NMSC while 24 KTR suffered from recurrent NMSC. **Figure 6** reveals differences in the percentages of CD8^+^ Treg and Tresp subsets between those two patient groups.

Compared to KTR without recurrent NMSC, those with NMSC recurrence revealed a significantly decreased percentage of naïve Tregs and an increased percentage of EM Tregs (**Figure 6A**). Additionally, the KTR with recurrent NMSC showed a decreased percentage of RTE Tregs and increased percentage of CD31^-^ memory Tregs (**Figure 6C**), suggesting increased RTE Treg differentiation into CD31^-^ memory Tregs independent of age. Similar changes were also implied in the Tresp compartment, but the decreased percentage of naïve Tresps and RTE Tresps in KTR with recurrent NMSC did not reach significance (**Figures 6C, D**). These results show that KTR with NMSC recurrence cannot intensify CM Treg/Tresp production and instead, shift their differentiation towards the production of EM Tregs/Tresps. Thereby, RTE Treg differentiation seems to be adequately intensified resulting in the decreased percentage of naïve and RTE Tregs in KTR with recurrent NMSC. Otherwise, RTE Tresp differentiation might rather be exhausted, as there is no significantly decreased percentage of naïve or RTE Tresps and only a slightly increased percentage of EM Tresps. These differences reflect a possibly reduced Tresp response in KTR when NMSC recurs.

## 4 Discussion

In healthy individuals, T cell immunity changes with age with an increase of Tregs and a corresponding decline of Tresps, causing an increasing Treg/Tresp ratio. These changes occur in both CD4^+^ and CD8^+^ T cells (18, 24), largely preventing autoimmune diseases, but increasing the susceptibility to infections and cancer in elderly individuals. Our former studies revealed that such increasing Treg/Tresp ratios are maintained by changing the differentiation pathways of RTE Tregs/Tresps with age so that in case of exhaustion of one pathway, another can be used. This ensures a sufficient differentiation of RTE T cells into memory T cells, where the additional differentiation of resting MN Tregs/Tresps is of particular importance, both maintaining age-dependent and age-independent enhanced differentiation in case of specific diseases (25, 26). Recently, we showed that chronic kidney failure provokes an age-independently increased differentiation of CD8^+^ RTE Tresps *via* pathway 1 (**Figure 3**), producing apoptosis-resistant CM Tresps which exhibited strong Fas ligand-mediated cytotoxicity. This differentiation was not maintained after transplantation so that the proportion of CM Tresps decreased strongly in KTR, while increased RTE Tresp proliferation preserved sufficient EM Tresp production. These age-independent changes also caused a reduced age-dependent resting MN Tresp differentiation into CM Tresps, but rather MN Tresp proliferation into EM Tresps instead. Similarly, RTE Treg differentiation into CM Tregs was strongly reduced in KTR. However, in contrast to Tresps, age-dependent RTE Treg differentiation into CM Tregs *via* pathway 1 was entirely restored in healthy KTR (18).

It is known that kidney failure comes along with a thymus involution accompanied by a reduced distribution of RTE Tregs/Tresps (17). This dysfunction may be restored after transplantation. Therefore, our current findings, revealing strongly enhanced differentiation of both CD8^+^ RTE Tregs and Tresps into CM cells in KTR developing *de-novo* NMSC, propose that the immunosuppressive therapy inhibits successive differentiation of freshly released RTE Tregs/Tresps sufficiently. This allows an intensified and effective immune response to tumor cells *via* pathway 1 when needed. CM Tresps secrete IFN-γ which facilitates cancer immunity by inhibiting tumor proliferation and angiogenesis (27, 28). Moreover, compared to EM and TEMRA Tresps, CM Tresps play a crucial role in Fas-L-induced apoptosis of cancer cells (18). The expression of Fas receptor on cancer cells is increased in the early phase of tumor growth (29, 30), which means that the increased occurrence of CM Tresps expressing membrane-bound Fas-L enables an early effective immune response to cancer. With age, the additional differentiation of resting MN Tregs/Tresps became necessary, where an increased conversion of these cells into CM Tregs could be maintained, while the production of resting MN Tresps and their subsequent conversion into CM Tresps ceased. Such differential regulation of the immune response during the course of life favors the development of NMSC, especially in older patients. The fact that resting MN Tregs/Tresps converted rather than proliferated into CD31^-^ memory Tregs/Tresps and that only Tresp differentiation *via* resting MN Tresps exhausted with age, provoke a strongly increased CD8^+^ Treg/Tresp ratio, especially in older KTR who develop NMSC. These findings propose this ratio as an excellent possible candidate for the identification of KTR at risk for NMSC in the high-risk group of older KTR.

Experiments using immunohistochemistry confirmed our results by finding an increased ratio of FoxP3^+^ Tregs to CD8^+^ T cells in NMSC tissue of transplant patients (31). Similar attempts were made to predict NMSC by the detection of increased numbers of CD4^+^ Tregs without correlating it with age-dependent CD4^+^ Treg/Tresp differentiation (32). Further hints that CD8^+^ T cell exhaustion or even immunosenescence is associated with post-transplant NMSC are given by the fact that CD57 expressing CD8^+^ T cells correlated with increasing CD8^+^ T cell differentiation and were found to predict the future occurrence of cutaneous squamous cell carcinoma (33). However, these studies refer to KTR with a history of cutaneous squamous cell cancer but did not exclusively investigate the prediction of *de-novo* cancer although it is known that a history of NMSC is a reliable clinical predictor for cancer recurrence. Therefore, a predictor for *de-novo* NMSC would be of greater importance. Presumably, transcriptional characterization, such as comparative scRNA analysis of CD8^+^ Tregs and CD8^+^ Tresps isolated from KTR who develop NMSC in the future, those who do not and those who are currently suffering from NMSC, could identify markers indicative of increased exhaustion, expressed on special CD8^+^ T cell subsets (34).

Meanwhile, several studies demonstrate the accumulation of exhausted and terminally differentiated CD8^+^ T cells in patients with solid cancers affecting the gastrointestinal tract and the lung (35–37). Thereby it seems to be important to distinguish between exhaustion and senescence of these cells, as exhaustion in contrast to senescence may be able to be reversed by blocking inhibitory receptors such as PD-1 (38). Recently, Peters et al. could demonstrate that differentially methylated regions in T cells identify KTR at risk for *de-novo* NMSC even before kidney transplantation (39), highlighting the importance of T cell response to cancer in immunosuppressed individuals.

In KTR already suffering from NMSC, the decreasing conversion of resting MN Tresps into CM Tresps could not be sufficiently compensated by increasing proliferation into EM Tresps and instead, favored a strong accumulation of CD31^-^ TEMRA Tresps with age. These findings may cause the frequent recurrence of NMSC, particularly in elderly patients. In the meantime, it is increasingly becoming clear that the majority of solid tumor-specific CD8^+^ T cells do not represent TEMRA cells but rather activated EM cells, exhibiting broad and intense T cell inhibitory receptor (TCIR) expression (PD-1, LAG-3, TIM-3, and TIGIT) (40), and that clonal expansion of these dysfunctional and exhausted cells occurs during disease progression (41, 42) and recurrence as our data show. On the other hand, tumor specific memory CD8^+^ T cells in an earlier differentiation status, such as CM cells were also shown to be present in solid tumors exhibiting superior anti-tumor activity, suggesting that a progenitor population is required to supply a sufficient anti-tumor response (43). Such findings suggest that CD8^+^ CM T cells may be the most potent memory subset for tumor immunity, while dysfunctional EM and TEMRA cells appear with tumor progression and recurrence, especially with increasing age.

However, our data also have limitations in that this study is exploratory, not confirmatory. In addition, we examined only CD8^+^ T cell differentiation in our patient groups, not CD4^+^ T cell differentiation, and patients who develop NMSC in the future might also have had an increased CD4^+^ Treg/Tresp ratio. The different differentiation pathways need further validation as we have not yet confirmed our assumptions by analyzing Ki67 expression of the different CD8^+^ T cell subsets (RTEs, MNs, CD31^+^ memory cells, CD31^-^ memory cells) in correlation with decreasing CD8^+^ RTE cells within naïve CD8^+^ T cells (pathway 1 and 2), or in correlation with decreasing CD8^+^ MN T cells within CD8^+^CD31^-^ T cells (pathway 3), as we have done for CD4^+^ T helper and Treg cells (25, 26). In order to functionally validate this theoretical model, it would have been necessary to stimulate a population *in vitro* and show that it could generate the next population. Further functional analysis of CD8^+^ Treg and Tresp cells needs to be performed to confirm the suppressive activity of CD8^+^ Tregs. In addition, the enhanced CD8^+^ Treg/Tresp response in KTR who develop NMSC in the future and the exhausted CD8^+^ Tresp phenotype in KTR who already suffer from NMSC need to be further confirmed. Another limitation of this study is the fact that absolute numbers of CD8^+^ T cells and their subsets were not determined. Future clinical trials should be conducted with a larger number of participants, as we were only able to recruit six KTR developing NMSC in the future. In this context, serial CD8^+^ T-cell analyses of individuals with an initially elevated CD8^+^ Treg/Tresp ratio would be of great importance to validate the elevated ratio as a suitable marker for *de novo* NMSC.

In KTR, the application of immunosuppressive therapy seems to be the most driving risk factor for the development of NMSC. Thereby, the tumor-promoting effect of immunosuppression is not only observed in transplant patients, but also in other diseases (rheumatoid arthritis, inflammatory bowel disease) in which immunosuppressive therapy is administered. Even patients with immunosuppressing systemic diseases, such as chronic lymphocyte leukemia, non-Hodgkin lymphoma, or HIV, have an increased risk of developing NMSC (44, 45). Therefore, it seems unlikely that immunosuppression exerts a major direct effect on tumor pathogenesis, such as inducing mutations or inhibiting DNA repair mechanisms. Rather, differential suppression of CD8^+^ Treg/Tresp differentiation and resulting differential exhaustion and dysfunction of CD8^+^ Tregs/Tresps appears to be decisive. It is discussed that excess immunosuppression promotes T cell senescence through repetitive reactivation of latent viruses (CMV, EBV, or papillomavirus), causing repeated inflammation and accumulation of oligoclonal expanded senescent T cells. However, since direct evidence is lacking, it seems more likely that the immunosuppressive therapy has a differential effect on tumor-promoting regulatory T cells and tumor-defending Tresp cells. Thereby, our results suggest a more inhibiting effect on Treg differentiation than on Tresp differentiation. Thus, in KTR without NMSC, the age-dependent differentiation of resting MN Tregs into functional CM Tregs can be maintained longer than that of resting MN Tresps into CM Tresps. In already diseased KTR, the exhausting proliferation of resting MN cells into dysfunctional EM Tresps and immunosenescent TEMRA Tresps, however to a lower degree into EM Tregs and TEMRA Tregs, may facilitate tumor progression. Therefore, it would be of great importance to develop new immunosuppressive therapies that can prevent such an imbalance in the differentiation of CD8^+^ regulatory and responder T cells.

## Data availability statement

The raw data supporting the conclusions of this article will be made available by the authors, without undue reservation.

## Ethics statement

The studies involving human participants were reviewed and approved by the ethics committee of the Medical Faculty of Heidelberg, University of Heidelberg, Heidelberg, Germany. The patients/participants provided their written informed consent to participate in this study.

## Author contributions

JL, MS, and AS designed the study. JL performed the study. MS and FK coordinated the sample collection. MZ contributed important patients. JL, MS, and AS analyzed the data and wrote the manuscript. All authors contributed to the article and approved the submitted version.
