# Supplementary material for: Exhaustion of CD8+ central memory responder T cell differentiation provokes non-melanoma skin cancer in elderly kidney transplant recipients
Source: Front Immunol. 2023 May 23;14:1164284. doi: 10.3389/fimmu.2023.1164284 (PMC10242110; doi:10.3389/fimmu.2023.1164284)
Supplement: Supplementary file 1 [file Table_1.docx]

| **Supplementary Table 1:** R^2^ coefficients of determination of the linear regressions. | | | |
| --- | --- | --- | --- |
|  | **Healthy KTR** | **KTR with NMSC** | **KTR developing NMSC** |
| Figure 2 |  |  |  |
| (A) Naïve Tregs | 0.3114 | 0.4859 | 0.3607 |
| (B) CM Tregs | 0.2442 | 0.2692 | 0.7008 |
| (C) TEMRA Tregs | 0.0318 | 0.1492 | 0.1114 |
| (D) EM Tregs | 0.1598 | 0.1753 | 0.2518 |
| (E) CD31^+^ TEMRA Tregs | 0.0118 | 0.0883 | 0.2914 |
| (F) RTE Tregs | 0.3728 | 0.5390 | 0.3435 |
| (G) CD31^+^ memory Tregs | 0.0312 | 0.1896 | 0.6070 |
| (H) CD31^-^ TEMRA Tregs | 0.0503 | 0.1749 | 0.2316 |
| (I) MN Tregs | 0.1940 | 0.0079 | 0.1212 |
| (J) CD31^-^ memory Tregs | 0.2998 | 0.2264 | 0.1128 |
| Figure 3 |  |  |  |
| (A) Naïve Tresps | 0.3305 | 0.4967 | 0.5150 |
| (B) CM Tresps | 0.0470 | 0.0645 | 0.2726 |
| (C) TEMRA Tresps | 0.3027 | 0.4487 | 0.3353 |
| (D) EM Tresps | 0.0328 | 0.0406 | 0.1627 |
| (E) CD31^+^ TEMRA Tresps | 0.2037 | 0.3741 | 0.3728 |
| (F) RTE Tresps | 0.3366 | 0.4973 | 0.4787 |
| (G) CD31^+^ memory Tresps | 0.0798 | 0.0797 | 0.1614 |
| (H) CD31^-^ TEMRA Tresps | 0.2681 | 0.3228 | 0.0676 |
| (I) MN Tresps | 0.0383 | 0.1596 | 0.4781 |
| (J) CD31^-^ memory Tresps | 0.1935 | 0.0801 | 0.2479 |
| Figure 5 |  |  |  |
| (A) CD8^+^ Tregs | 0.2954 | 0.1542 | 0.3172 |
| (B) CD8^+^ Tresps | 0.2954 | 0.1542 | 0.3172 |
| (C) CD8^+^ Treg/Tresp ratio | 0.2966 | 0.1546 | 0.3161 |
| (D) CD8^+^ T cells | 0.1143 | 0.2778 | 0.5711 |
| *CM, central memory; EM, effector memory; KF, kidney failure; MN, mature naïve; TEMRA, terminally differentiated effector memory; Treg, regulatory T cell; Tresp, responder T cell* | | | |
